# Bipolar stacked quasi-all-solid-state lithium secondary batteries with output cell potentials of over 6 V

**DOI:** 10.1038/srep06084

**Published:** 2014-08-15

**Authors:** Takahiro Matsuo, Yoshiyuki Gambe, Yan Sun, Itaru Honma

**Affiliations:** 1 Institute of Multidisciplinary Research for Advanced Materials, Tohoku University, 2-1-1 Katahira, Aoba-ku, Sendai, Miyagi 980-8577, Japan

## Abstract

Designing a lithium ion battery (LIB) with a three-dimensional device structure is crucial for increasing the practical energy storage density by avoiding unnecessary supporting parts of the cell modules. Here, we describe the superior secondary battery performance of the bulk all-solid-state LIB cell and a multilayered stacked bipolar cell with doubled cell potential of 6.5 V, for the first time. The bipolar-type solid LIB cell runs its charge/discharge cycle over 200 times in a range of 0.1–1.0 C with negligible capacity decrease despite their doubled output cell potentials. This extremely high performance of the bipolar cell is a result of the superior battery performance of the single cell; the bulk all-solid-state cell has a charge/discharge cycle capability of over 1500 although metallic lithium and LiFePO_4_ are employed as anodes and cathodes, respectively. The use of a quasi-solid electrolyte consisting of ionic liquid and Al_2_O_3_ nanoparticles is considered to be responsible for the high ionic conductivity and electrochemical stability at the interface between the electrodes and the electrolyte. This paper presents the effective applications of SiO_2_, Al_2_O_3_, and CeO_2_ nanoparticles and various Li^+^ conducting ionic liquids for the quasi-solid electrolytes and reports the best ever known cycle performances. Moreover, the results of this study show that the bipolar stacked three-dimensional device structure would be a smart choice for future LIBs with higher cell energy density and output potential. In addition, our report presents the advantages of adopting a three-dimensional cell design based on the solid-state electrolytes, which is of particular interest in energy-device engineering for mobile applications.

Renewable energy sources, which unlike exhaustible energy sources such as petroleum or natural gas, do not generate carbon dioxide that is considered the cause of global warming, are attracting considerable attention. Renewable energy sources, which exist in nature, are expected to provide clean energy, such as solar energy, wind energy, tidal energy, and geothermal energy. Energy storage devices that can store energy efficiently are essential for utilizing renewable energy. Lithium ion batteries (LIBs) with high energy density are an example of such devices and have attracted significant attention in recent times. LIBs are currently used not only for compact applications, e.g., as power sources for electronic devices but also for larger applications such as in electric vehicles and stationary power sources. Conventional LIBs use organic liquid electrolytes, and there is possibility of liquid leaks and dangers such as ignition. Such problems must be resolved for practical and safe use of LIBs. The use of all-solid-state secondary batteries that use solid electrolytes, which are flame resistant and do not pose the risk of liquid leaks, can be cited as a possible solution to these problems. In addition to the fact that there is no possibility of liquid leaks or dangers of ignition, all-solid-state lithium secondary batteries make it possible to design bipolar layer-built cells fabricated by layering batteries within a single package. The energy density in such batteries is expected to be higher than that in LIBs with organic liquid electrolytes. However, solid electrolytes used in all-solid-state secondary batteries pose certain issues. For instance, solid electrolytes that have sufficient ion conductivity and high stability when used with lithium metal electrode are not abundantly available, and it is difficult to achieve a good contact between the solid electrolytes and cathode materials^1^. In order to resolve such issues, our research group has been investigating the use of quasi-solid-state electrolytes. These materials are prepared by solidifying lithium-ion-conductive ionic liquids that are flame resistant and have high ionic conductivity, by utilizing the strong interaction on the surface of oxide nanoparticles^2^,^3^,^4^,^5^. Quasi-solid-state electrolytes that contain SiO_2_ nanoparticles (particle diameter: 7 nm) as oxide nanoparticles and cation-bis(trifluoromethanesulfonyl)amide(TFSA)/Li-TFSA (cation: 1-ethyl-3-methyl imidazolium (EMI), *N,N*-diethyl-*N*-methyl-*N*-(2-methoxyethyl) ammonium (DEME), *N*-methyl-*N*-propyl piperidinium (PP13)) as lithium-ion-conducting ionic solutions, have mechanical strength as well as transport properties similar to those of liquids; moreover, their electrical conductivity and self-diffusion coefficient are also not significantly different from those of liquids^5^. Furthermore, all-solid-state lithium batteries fabricated using such quasi-solid-state electrolytes have been confirmed to operate favorably at 0.1 C. We also considered quasi-solidifying glyme-lithium salt complexes ([Li (G4)] [TFSA]) having characteristics similar to those of ionic liquids, as reported by Watanabe and associates^6^,^7^,^8^,^9^,^10^. [Li (G4)] [TFSA] has been reported to be electrochemically stable up to 0 V (vs. Li/Li^+^) and has relatively high electrical conductivity. Further, the quasi-solid-state electrolyte containing [Li (G4)] [TFSA] and SiO_2_ nanoparticles, which has transport characteristics similar to those of [Li (G4)] [TFSA] liquids, operates favorably in all-solid-state secondary batteries^11^. However, when constant polarization measurements for symmetrical cells fabricated using Li metal were conducted, quasi-solid electrolytes containing [Li (G4)] [TFSA] indicated spike-wave amperometric responses under relatively small voltages^11^. This is believed to have occurred because of electrolyte penetration due to dendrite precipitation. Moreover, the electrical conductivity of these electrolytes needs to be improved further. It is necessary to inhibit dendrite precipitation and to design quasi-solid-state electrolytes with higher electrical conductivity for using them in all-solid-state secondary batteries. We attempted to design quasi-solid electrolytes with high stability to Li metal and excellent transport characteristics, by creating favorable boundaries between ionic solutions and oxide nanoparticles. The electrolytes were prepared using Al_2_O_3_ andCeO_2_ nanoparticles that are different from SiO_2_ nanoparticles that are currently used for this purpose.

## Results

### Fabrication of quasi-solid-state electrolyte

**Figure 1:**
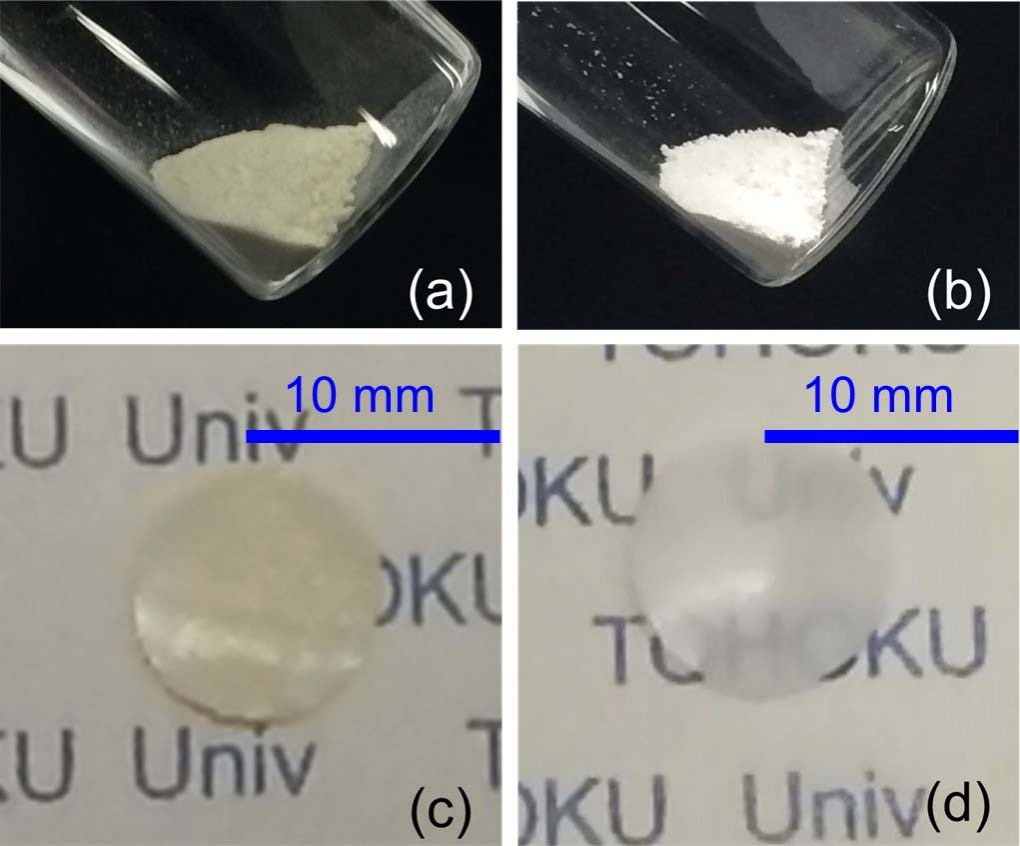
Photographs of quasi-solid state composite powders prepared using (a) CeO_2_ and (b) γ-Al_2_O_3_, and 200-µm-thick electrolyte self-standing sheets prepared using (c) CeO_2_ and (d) γ-Al_2_O_3_.

**Figure 2:**
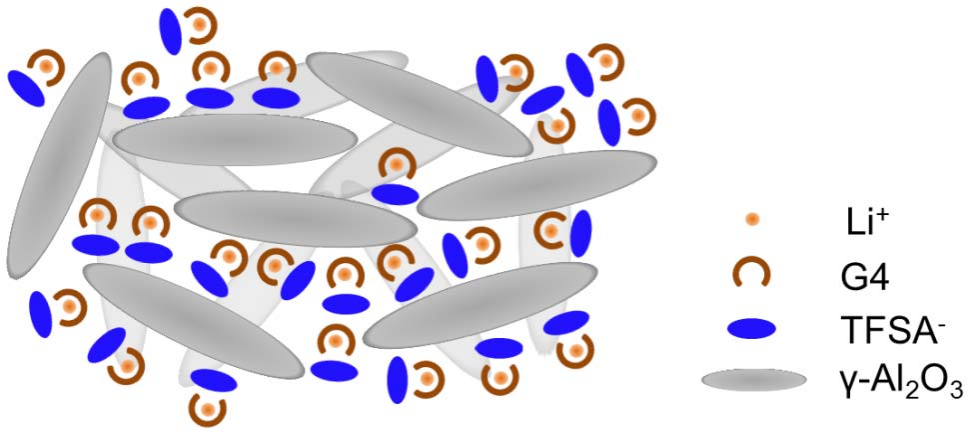
Schematic of QSE containing [Li (G4)] [TFSA] and γ-Al_2_O_3_.

**Figure 3:**
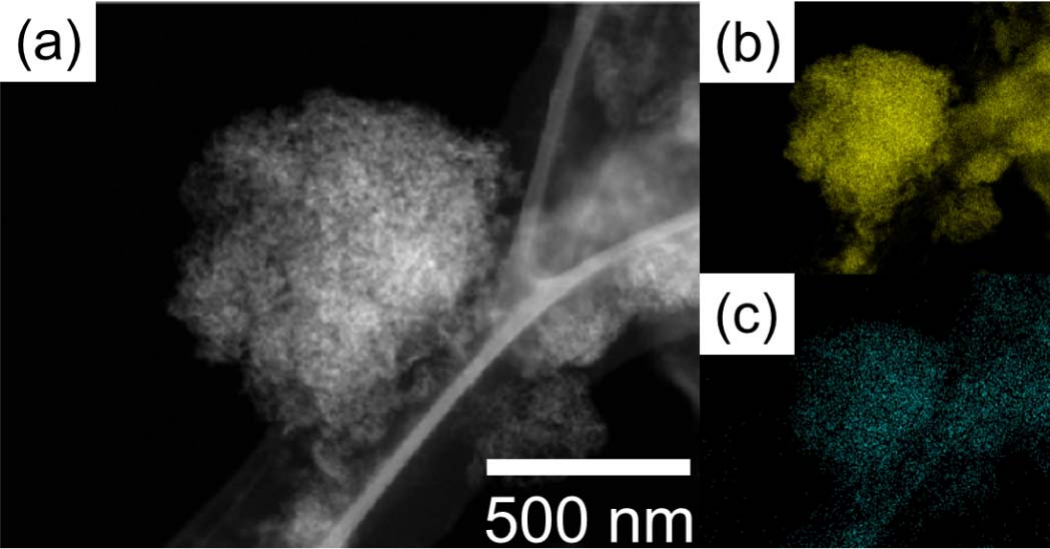
TEM image and Al and F distributions of QSE containing [Li (G4)] [TFSA] and γ-Al_2_O_3_; (a) TEM image and elemental mapping of (b) Al and (c) F.

In this work, we defined a quasi-solid-state electrolyte as the one that can be treated as a solid but that can maintain liquid-like high ionic conductivity. Results for the preparation of the quasi-solid-state electrolytes prepared using different oxide nanoparticles at respective volume fractions are listed in Table 1. In cases when SiO_2_ was used, it was possible to fabricate quasi-solid-state electrolytes that contain composites of [Li (G4)] [TFSA] and oxide nanoparticles with *x* = 75, where *x* is the volume fraction of the composites as reported earlier^11^. On the other hand, in the case of γ-Al_2_O_3_, α-Al_2_O_3_, and ZrO_2_ with particle diameters of up to 20 nm, 50 nm, and 10 and 5 nm, respectively, although quasi-solidification was possible up to *x* = 60 owing to interactions between oxide nanoparticle surfaces and [Li (G4)] [TFSA] liquids, no quasi-solidification was possible at *x* = 75. Quasi-solidification was possible at *x* = 75 with particle diameters of 10–30 nm for CeO_2_ and 5 nm for γ-Al_2_O_3_. Thus, although quasi-solidification was not possible at *x* = 75 with a particle diameter of 20 nm for γ-Al_2_O_3_, it was possible with a particle diameter of 5 nm. This is believed to be because when particle diameters are small, the specific surface area becomes greater and the interaction area between the oxide nanoparticle surfaces and [Li (G4)] [TFSA] liquids becomes larger. The quasi-solid electrolyte powder and free-standing film sheets fabricated using CeO_2_ with particle diameters of 10–30 nm and using γ-Al_2_O_3_ with a particle diameter of 5 nm are shown in Figure 1. Further, a schematic depicting the hypothesized conditions for [Li (G4)] [TFSA] and γ-Al_2_O_3_ nanoparticles in quasi-solid-state electrolytes at *x* = 75 is shown in Figure 2. The liquid and oxide nanoparticles in quasi-solid electrolytes as observed using TEM are shown in Figure 3. The images confirmed that Al (indicating γ-Al_2_O_3_) and F (indicating [Li (G4)] [TFSA]) were evenly distributed. The electrochemical properties of quasi-solid-state electrolytes fabricated using CeO_2_ with particle diameters of 10–30 nm and using γ-Al_2_O_3_ with a particle diameter of 5 nm at *x* = 75 are described below.

### Electrical conductivity measurements by alternating current impedance method

**Figure 4:**
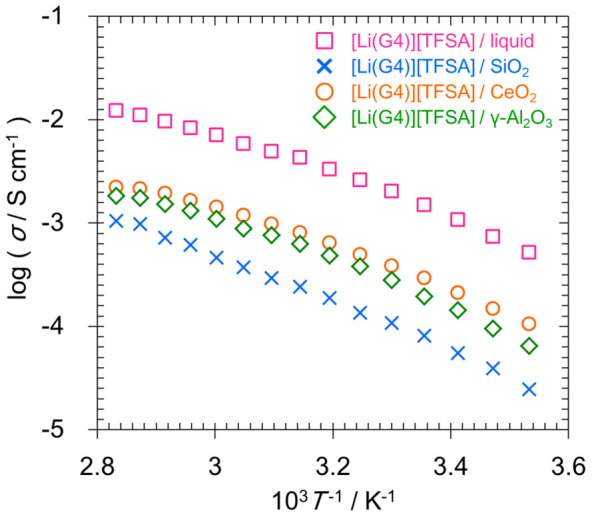
Ionic conductivities of quasi-solid-state electrolytes prepared using SiO_2_, CeO_2_, γ-Al_2_O_3_, and [Li (G4)] [TFSA].

The results of the electrical conductivity measurement are shown in Figure 4. Electrical conductivities of liquid [Li (G4)] [TFSA] and of quasi-solid-state electrolytes that are prepared using SiO_2_, CeO_2_, and γ-Al_2_O_3_ and that can be treated as solids when the volume fraction of the [Li (G4)] [TFSA] liquid is 75 vol%, are shown. The electrical conductivity of quasi-solid electrolytes of CeO_2_ and γ-Al_2_O_3_ prepared in this study was lower than that of the [Li (G4)] [TFSA] liquid but was higher than that of the [Li (G4)] [TFSA]/SiO_2_ quasi-solid-state electrolyte that was previously reported^11^. CeO_2_ interacts with cations and anions and has been reported to have significant effects on the transference number and electrical conductivity^12^,^13^. Furthermore, it has also been reported that by adding an appropriate amount of γ-Al_2_O_3_ particles, the electrical conductivity of Li^+^-conducting polymer electrolytes can be improved and favorable interfaces with Li metal can be realized^14^. Therefore, such characteristics of nanoparticles could be shown by the quasi-solid-state electrolytes prepared in this study. Further, γ-Al_2_O_3_ and CeO_2_ are considered to have transport characteristics that resemble those of liquids, since the temperature dependence of electrical conductivity of the [Li (G4)] [TFSA] bulk did not change owing to quasi-solidification.

### Constant polarization measurements

**Figure 5:**
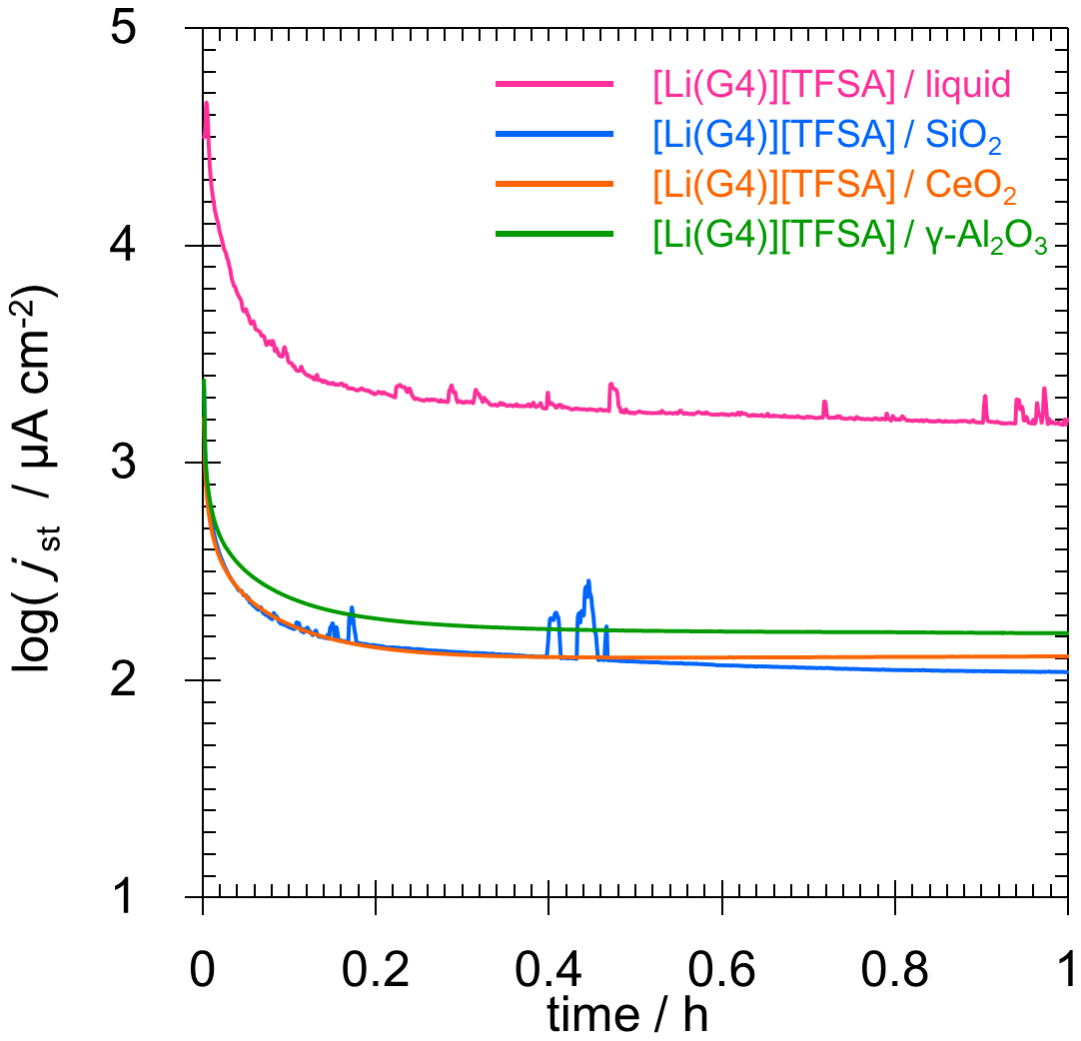
Typical current profiles of lithium symmetric cells at an applied voltage of 700 mV.

The results of constant polarization measurements for the Li symmetrical cell are shown in Figure 5. The currents reached a steady state at applied voltages below 500 mV in the case of all quasi-solid-electrolytes. On the other hand, a spike-wave amperometric response was indicted by the [Li (G4)] [TFSA]/SiO_2_ quasi-solid-state electrolytes and [Li (G4)] [TFSA] liquid when a voltage of 700 mV was applied. This is believed to be due to the separator as well as penetration of the quasi-solid electrolytes by dendrite precipitation^15^,^16^,^17^,^18^. Quasi-solid-state electrolyte sheets fabricated using CeO_2_ and γ-Al_2_O_3_ particles, on the other hand, indicated no spike-wave amperometric responses and were able to supply steady-state currents. This is believed to be because the use of CeO_2_ and γ-Al_2_O_3_ particles increased the mechanical strength to levels that exceeded that of conventional quasi-solid electrolytes prepared using SiO_2_, and this made it possible to use the Li metal negative electrode in a more stable manner.

### Fabrication of all-solid-state lithium secondary batteries, cross-sectional SEM observations of all-solid-state cell, and charge-discharge measurements

**Figure 6:**
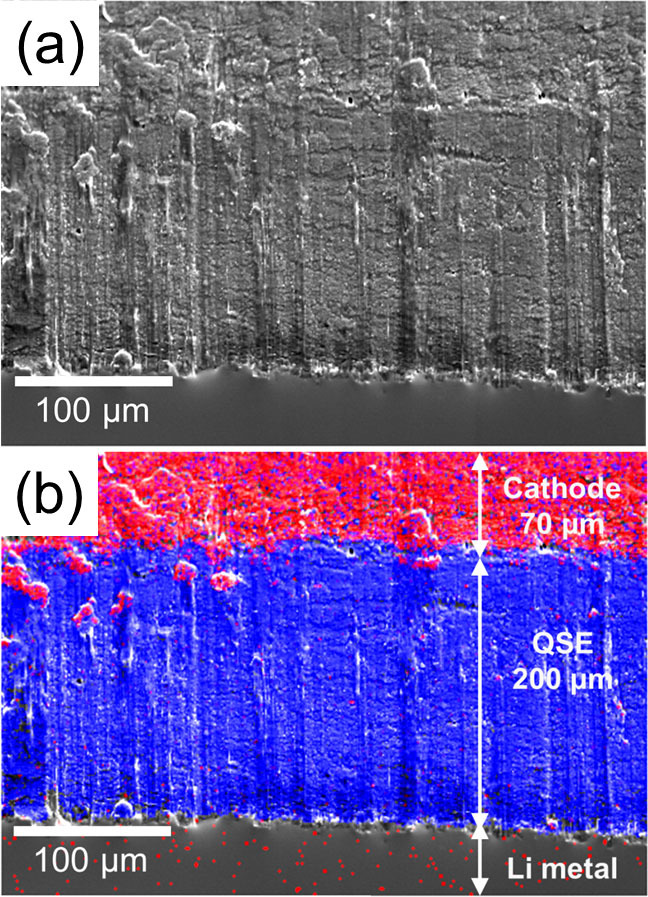
Cross-sectional SEM image and X-ray elemental mapping of P and Al for all-solid-state lithium battery; (a) SEM image and (b) elemental mapping of P (red) and Al (blue).

The SEM observation results for the cross sections of the fabricated all-solid-state battery using quasi-solid-state electrolyte prepared using γ-Al_2_O_3_ are shown in Figure 6. Red and blue represent P and Al, respectively, and each of these elements is derived from LiFePO_4_ in the cathode composite and γ-Al_2_O_3_ in the quasi-solid electrolyte. The cross-sectional SEM images confirmed that continuous dense interfaces were formed between the surface of the cathode composite and quasi-solid-state electrolyte.

**Figure 7:**
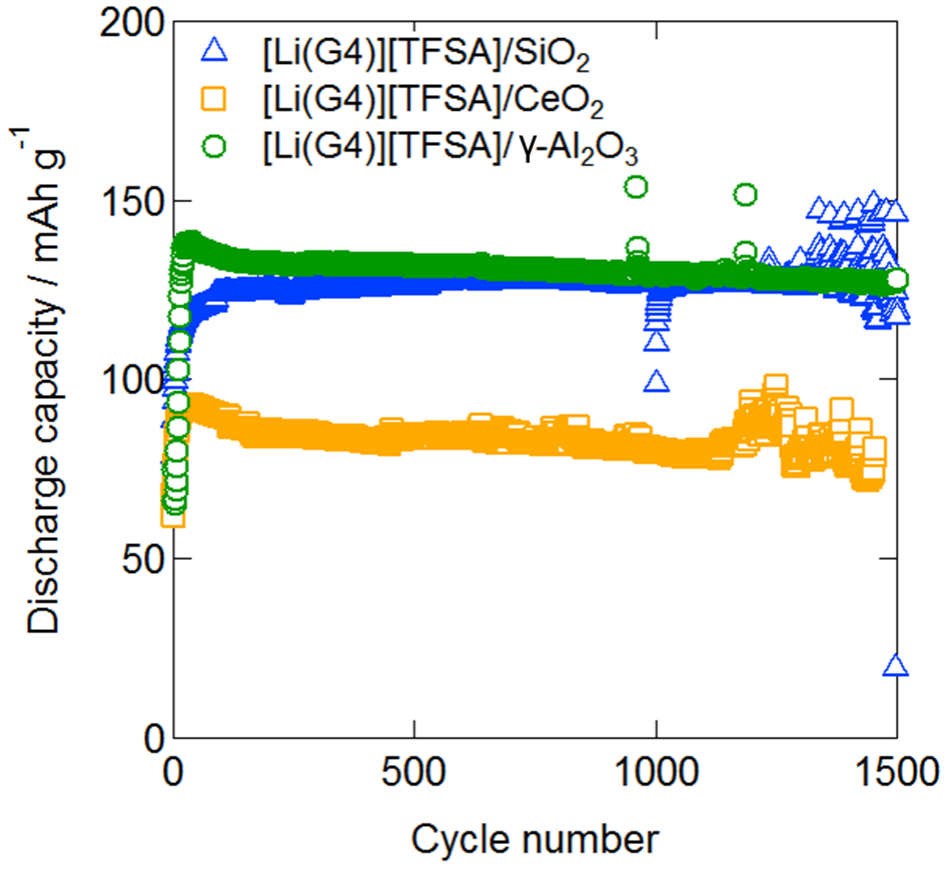
Cycle performance of all-solid-state batteries at 308 K and 1.0 C with electrolytes prepared using SiO_2_, CeO_2_, and γ-Al_2_O_3_.

**Figure 8:**
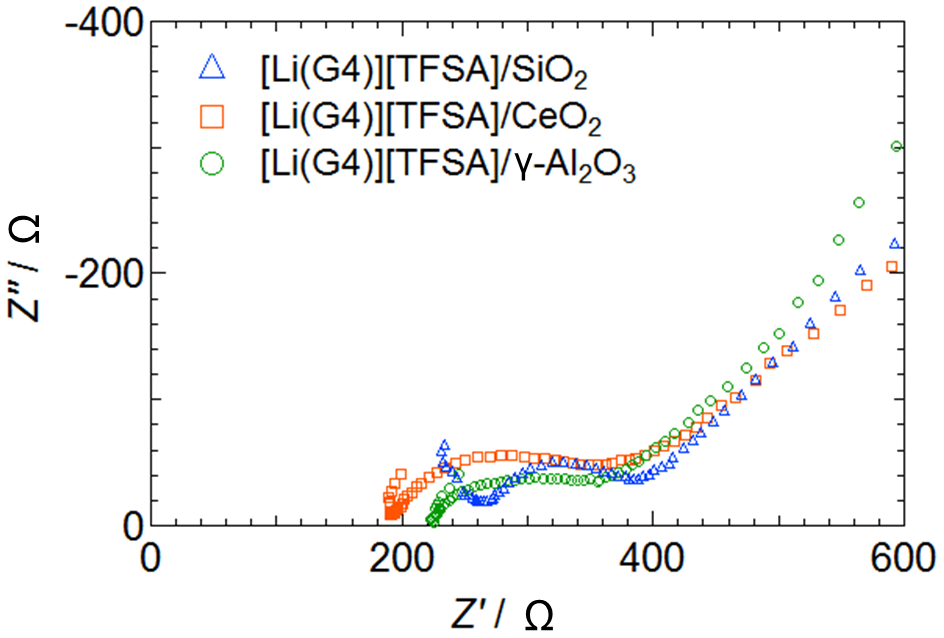
Cole-Cole plots of ac impedance measurement for quasi-solid-state electrolytes prepared using SiO_2_, CeO_2_, and γ-Al_2_O_3 _sandwiched between cathode composites at 308 K.

The cycle characteristics of the single-layer all-solid-state lithium secondary battery at 1.0 C are shown in Figure 7. No capacity degradation occurred up to 1,000 cycles and electric discharge capacity was maintained for all quasi-solid-state electrolytes composed of any oxide nanoparticles. The cathode utilization rate of devices that used [Li (G4)] [TFSA]/SiO_2_, [Li (G4)] [TFSA]/CeO_2_, and [Li (G4)] [TFSA]/γ-Al_2_O_3_ was 74%, 47%, and 76%, respectively. Results indicating high conductivity for [Li (G4)] [TFSA]/CeO_2_ quasi-solid-state electrolytes led us to anticipate an increase in the discharge capacity owing to the cathode utilization rate being higher than that for other systems. However, the actual results were different from those anticipated. The resistance of the interface between the cathode composite and the quasi-solid-state electrolyte is considered one of the reasons for this difference in results. Figure 8 shows impedance spectra of the quasi-solid-state electrolyte sandwiched between cathode composites prepared using SiO_2_, CeO_2_, and γ-Al_2_O_3_. The values of charge transfer resistance in the case of SiO_2_, CeO_2_, and γ-Al_2_O_3_ were 120, 177, and 127, respectively. Although the electric charge transfer resistance for the interfaces of quasi-solid-state electrolytes and cathode composites prepared using SiO_2_ and γ-Al_2_O_3_ were almost equal, the electric charge transfer resistance for the interfaces with CeO_2_ was higher. Although it is difficult to draw conclusions regarding these observations, we believe that the higher charge transfer resistance can be considered the most probable reason.

**Figure 9:**
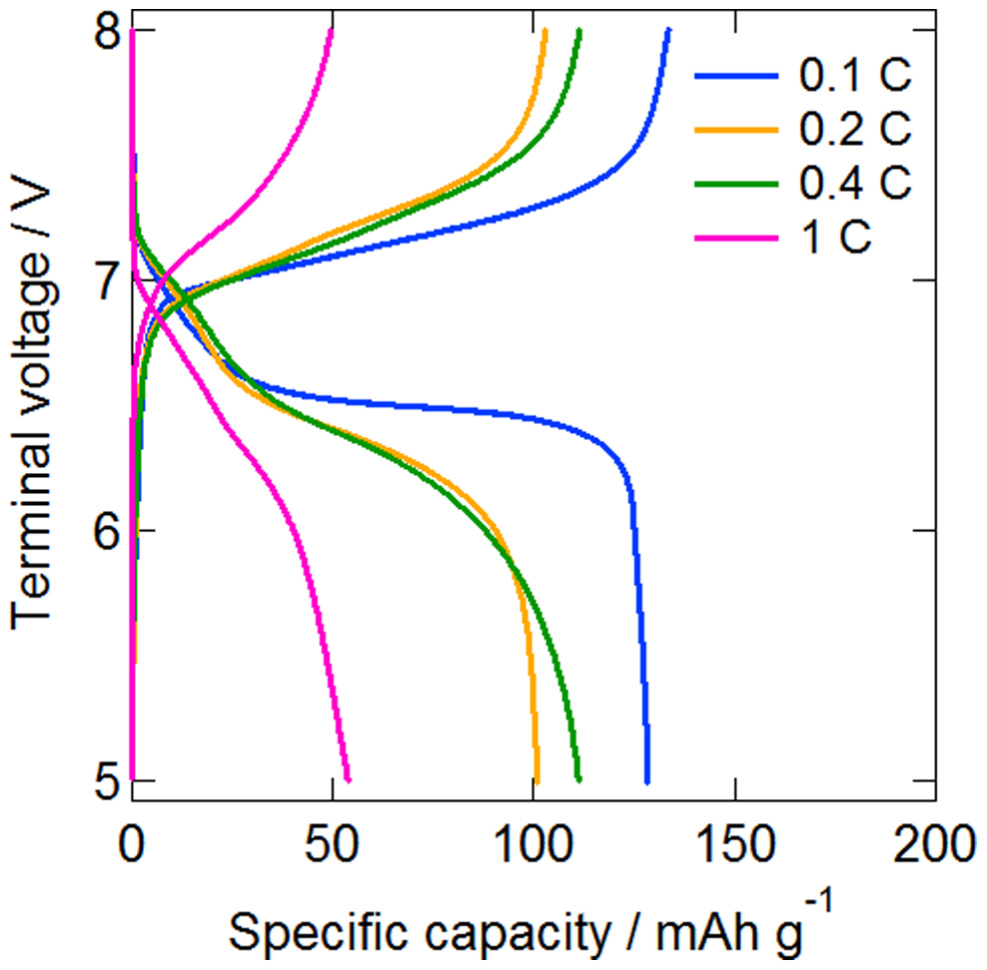
Charge–discharge profiles of bipolar stacked (double layer) all-solid-state batteries after 50 cycles at 308 K and 0.1, 0.2, 0.4, and 1.0 C.

**Figure 10:**
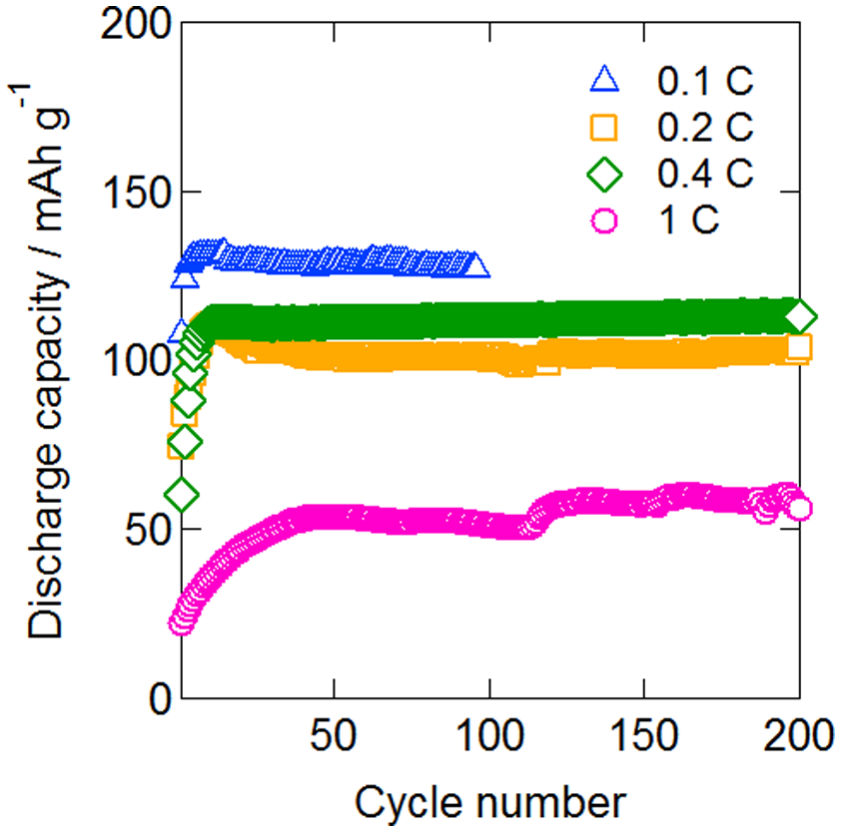
Cycle performance of bipolar stacked (double-layer) all-solid-state batteries at 308 K and 0.1, 0.2, 0.4, and 1.0 C.

The electric charging and discharging profiles of the double-layer all-solid-state lithium secondary battery fabricated using [Li (G4)] [TFSA]/γ-Al_2_O_3_ quasi-solid-state electrolytes after 50 cycles are shown in Figure 9. Currents of 128, 101, 111, and 54 mAh g^−1^ were observed for rates of 0.1, 0.2, 0.4, and 1.0 C, respectively, with the coulombic efficiency being 96% or more in all cases. Further, electric discharging plateaus were observed from 6.5 to 6.6 V, 6.3 to 6.5 V, 6.2 to 6.5 V, and 6.0 to 6.4 V, respectively. The plateau electrical potentials were observed to be double of those for the single-layer type, which ranged from 3.0 to 3.3 V, indicating that the layered cell was operating well without any short-circuiting of the single cell inside the single package. The packaging energy densities of single and double cell are 122 mWh/kg-cell and 176 mWh/kg-cell, respectively. The bipolar stacked cell thus had a higher energy density. The electric discharge capacity at 1.0 C and 50 cycles was found to be 137 mAh g^−1^ with the single-layer type, while that of the double-layer type was significantly lower at 54 mAh g^−1^. This difference was attributed to the application of the impregnation process. Favorable interfaces with a small number of pores were formed with quasi-solid-state electrolytes of the single-layer type, while the lack of the impregnation process in the case of the quasi-solid-state electrolytes of the double-layer type made the formation of favorable electrical conductivity paths difficult. The cycle characteristics of the double-layer all-solid-state lithium secondary battery at various C rates are shown in Figure 10. Favorable operation with hardly any capacity deterioration was observed at all C rates, even beyond 100 cycles.

## Discussion

In this study, [Li (G4)] [TFSA] quasi-solid-state electrolytes were prepared using various oxide nanoparticles and electrochemical characteristic evaluations and device evaluations were conducted. Quasi-solid-state electrolytes using CeO_2_ and γ-Al_2_O_3_ showed improved transport characteristics, facilitating a more stable use of a Li negative electrode than quasi-solid-state electrolytes prepared using SiO_2_, details for which have already been reported in the past. Furthermore, single-layer all-solid-state lithium secondary batteries using such quasi-solid-state electrolytes discharged in a stable manner even after 1,000 cycles. Favorable operation was observed for the double-layer all-solid-state secondary batteries with the quasi-solid-state electrolytes prepared using γ-Al_2_O_3_. The quasi-solid-state electrolytes prepared in this study are expected to broaden device design guidelines.

## Methods

### Fabrication of quasi-solid electrolyte and TEM observations

A solution was prepared by mixing lithium bis(trifluoromethanesulfonyl)amide powder (Li-TFSA; purity > 99%; Kishida Chemical Co., Ltd.) as a lithium salt to become equimolar in tetraethylene glycol dimethyl ether (G4; purity: 99%; Sigma Aldrich Co.). This [Li (G4)] [TFSA] solution was mixed with seven types of oxide nanoparticles at a volume fraction *x* = 40, 50, 60, and 75 vol% in methanol by stirring for 3 h. The mixed solution was then dried for 12 h at 60°C on a hot plate to evaluate the fabrication of quasi-solid-state electrolytes (QSE). The seven types of oxide nanoparticles were as follows: SiO_2_ (Sigma-Aldrich Co.; particle diameter: 7 nm), CeO_2_ (Kanto Chemical Co., Inc.; particle diameter: 10–30 nm), γ-Al_2_O_3_ (Kanto Chemical Co., Inc.; particle diameter: 5 nm), γ-Al_2_O_3_ (Kanto Chemical Co., Inc.; particle diameter: 20 nm), α-Al_2_O_3_ (Kanto Chemical Co., Inc.; particle diameter: 50 nm), ZrO_2_ (Kanto Denka Kogyo Co., Ltd.; particle diameter: 5 nm) and ZrO_2_ (Kanto Denka Kogyo Co., Ltd.; particle diameter: 10 nm). The free-standing films were prepared by mixing the quasi-solid-state powders and 5 wt% polytetrafluoroethylene (PTFE, Teflon-J, DuPont-Mitsui Fluorochemicals Co., Ltd.) in an agate mortar. The whole preparation process was carried out in an argon-atmosphere glove box.

### Electrical conductivity measurements

The fabricated quasi-solid electrolytic sheets and ionic liquid bulk were trapped between SUS316L electrodes to fabricate coin cells (CR2032 type). These coin cells were used for carrying out electrical conductivity measurements by the alternating current impedance method. Measurements were carried out under the following conditions: temperature range, 10–80°C; electrical potential amplitude, 10 mV; and frequency range, 1 Hz–1 MHz. Ionic conductivity was calculated using the following equation with the measured resistance value *R_b_* [Ω]. 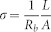
where L [cm] is the distance between the electrodes and A [cm^2^] is the area of the electrodes.

### Constant polarization measurements

Constant polarization measurements were conducted in order to evaluate the stability of the electrolytes with regard to Li metal. The Li metal sample was cut into 10-mm-diameter circular sheets, and the quasi-solid electrolyte sample was cut into 12-mm-diameter circular sheets. The electrolyte sheets were then trapped within the Li metal sheets to fabricate a symmetrical cell. Liquids were processed by impregnating propylene separators (Celgard® 3501, Celgard; film thickness: 25 µm; porosity: 55%), eight sheets that were stacked to form a 200-µm-thick layered structure and that trapped a Li metal sheet with a diameter of 16 mm, which is equal in size to the current collector of a coin cell. A CR2032-type coin cell was used as a symmetrical cell. The amperometric response of the cell was observed for 1 h for each voltage value applied, and the electric current at each point of time was considered constant. The applied voltage was increased gradually as follows: 2, 5, 10, 20, 30, 40, 50, 70, 100, 150, 200, 300, 400, 500, 700, and 1000 mV. In order to alleviate the precipitation of the Li metal due to a continuous flow of electric current in one direction, the voltage of the same magnitude was applied in the reverse direction and measurements were conducted in an identical manner for each step.

### Fabrication of all-solid-state lithium secondary batteries and all-solid-state cell, cross-sectional SEM observations, and electric discharge measurements

LiFePO_4_ (LFP, Tatung Fine Chemicals Co.; theoretical capacity: 170 mAh g^−1^), acetylene black (AB), quasi-solid electrolyte powder, and PTFE were mixed with weight fractions of 34:11:45:10, respectively, to prepare a cathode composite from which a sheet with a diameter of 7 mm was fabricated. Single-layer quasi-all-solid-state lithium secondary batteries were prepared by directly stacking cathode composite, QSE sheet with a diameter of 12 mm and a Li metal anode with a diameter of 10 mm without any further treatment. The cathode composite and the QSE sheet were immersed in a [Li (G4)] [TFSA] solution for 30 min and subjected to the vacuum drying process. A double-layer all-solid-state battery was also fabricated by layering two single-layer solid batteries and trapping them with an electrical current collector SUS304L inside the same module as the CR2032 coin cell. No impregnation of the electrolyte sheets was performed for the double-layer battery. The cut off voltages were set to 2.0 to 4.0 V for the single-layer battery and 5.0 to 8.0 V for the double-layer battery. The electric discharge measurements were conducted at 35°C by the two-terminal method. Furthermore, the layered batteries were cut and their cross sections were observed using scanning electron microscopy (SEM, JSM-7001F, JEOL) and energy-dispersive X-ray spectrometry (EDS, Inca x-act, Oxford Instruments).

## Author Contributions

T.M. and I.H. conceived and designed this work. T.M., Y.G., and Y.S. carried out the synthetic experiments and conducted the electrochemical test. T.M. wrote the paper; all the authors participated in analysis and discussion of the results.

## Figures and Tables

**Table 1:** Quasi-solidification of [Li (G4)] [TFSA] with various oxide nanoparticles in *x* vol% (*x* = 40, 50, 60, 75) (QSE = quasi-solid state electrolyte)

	Diameter/nm	40 vol%	50 vol%	60 vol%	75 vol%
SiO_2_	7	QSE	QSE	QSE	QSE
CeO_2_	10-30	QSE	QSE	QSE	QSE
γ-Al_2_O_3_	5	QSE	QSE	QSE	QSE
	20	QSE	QSE	QSE	Gel
α-Al_2_O_3_	50	QSE	QSE	QSE	Gel
ZrO_2_	5	QSE	QSE	QSE	Gel
	10	QSE	QSE	QSE	Gel
